# A suite of web applications to streamline the interdisciplinary collaboration in secondary data analyses

**DOI:** 10.1186/1471-2288-4-29

**Published:** 2004-12-14

**Authors:** Ricardo Pietrobon, Ulrich Guller, Henrique Martins, Andreia P Menezes, Laurence D Higgins, Danny O Jacobs

**Affiliations:** 1 Center for Excellence in Surgical Outcomes, Duke University Medical Center, Duke University, Durham, NC, USA; 2 Center for Excellence in Surgical Outcomes, University Hospital Basel, Department of General Surgery and Surgical Research, Basel, Switzerland; 3 Duke University Health System, Rio Claro/Sao Paulo, Brazil; 4 Reliable International Research, Center for Excellence in Surgical Outcomes, Campina Grande do Sul/ Parana – Brazil; 5 Department of Surgery, Duke University Medical Center, Durham, NC, USA; 6 Department of Internal Medicine, University of North Carolina at Chapel Hill, NC, USA

**Keywords:** biomedical research, statistical data interpretation, research design, planning techniques, Internet, computer-assisted instruction

## Abstract

**Background:**

We describe a system of web applications designed to streamline the interdisciplinary collaboration in outcomes research.

**Description:**

The outcomes research process can be described as a set of three interrelated phases: design and selection of data sources, analysis, and output. Each of these phases has inherent challenges that can be addressed by a group of five web applications developed by our group. QuestForm allows for the formulation of relevant and well-structured outcomes research questions; Research Manager facilitates the project management and electronic file exchange among researchers; Analysis Charts facilitate the communication of complex statistical techniques to clinicians with varying previous levels of statistical knowledge; Literature Matrices improve the efficiency of literature reviews. An outcomes research question is used to illustrate the use of the system.

**Conclusions:**

The system presents an alternative to streamline the interdisciplinary collaboration of clinicians, statisticians, programmers, and graduate students.

## Background

In the last decade, the number of relevant data sources available for outcomes research has grown exponentially. In contrast, the number of individual researchers with clinical and statistical expertise required to explore these data sets increase at a much slower pace. As a result, an immense quantity of valuable clinical data are left untouched, never becoming clinical publications that could potentially improve health care.

The disproportion between data volume and number of qualified researchers can be explained by the growing complexity involved in outcomes research projects using secondary data analyses. Researchers have to formulate of a clinically relevant and methodologically sound research question, find appropriate data sources, perform statistical analyses, and generate a final manuscript that will be submitted for peer-review. Frequently, individual researchers have the training and time to perform a few of these steps, but the integration of all tasks calls for an interdisciplinary systems approach [1,2]. This interdisciplinary effort, however, is often challenged by communication problems among researchers with different backgrounds, particularly when physicians with an exclusive clinical education attempt to work in collaboration with quantitative researchers such as statisticians [3]. As a consequence, the output of such collaboration is either scarce or absent.

This article describes a suite of web applications developed to facilitate the process of converting outcome databases into clinical manuscripts, to streamline the interdisciplinary collaboration of researchers, and to connect all different steps of the outcomes research process. To illustrate its use, we will describe how a research project has been conducted using this system from its early phase of research question formulation to the completion of the final manuscript.

## Construction and content

**Figure 1:**
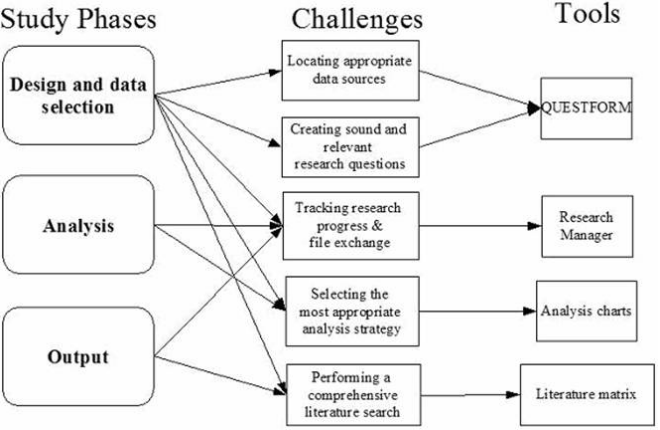
Research phases, challenges, and respective tools

The system of Web applications is composed by five different tools: QuestForm, Research Manager, Analysis Charts, and Literature Matrices. These tools were designed to assist researchers in each of the phases encountered in an outcomes research project involving secondary data analysis (Figure 1). All tools are freely available at a designated web site . The following sections will describe each of the Web applications and their application in the answer of a real outcomes research question.

### QuestForm

#### General description

**Figure 2:**
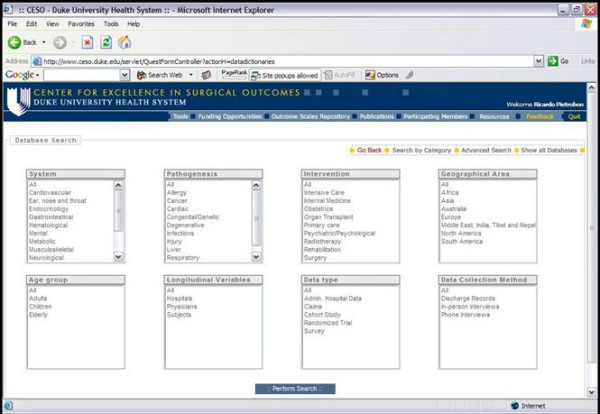
QuestForm application – Database search engine

QuestForm, an acronym for "Question Formulation", is an application designed to assist researchers in the location of clinical databases and formulation of outcome research questions (Figure 2). Clinical databases contain raw data (observations from individual patients) from national administrative claim data, cohort studies, clinical trials, and registries (see  for an updated list). All databases have been de-identified and do not contain protected health information as specified by the Health Insurance Portability and Accountability Act (, accessed on Aug/04/2004). The application The application is built using Extensible HyperText Markup Language 1.0 (XHTML) [4], Java, and a relational database (MySQL 4.0)[5].

**Figure 3:**
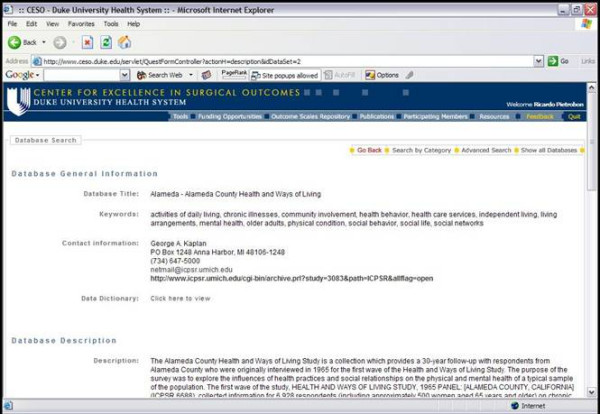
QuestForm application – Overall description of the database

**Figure 4:**
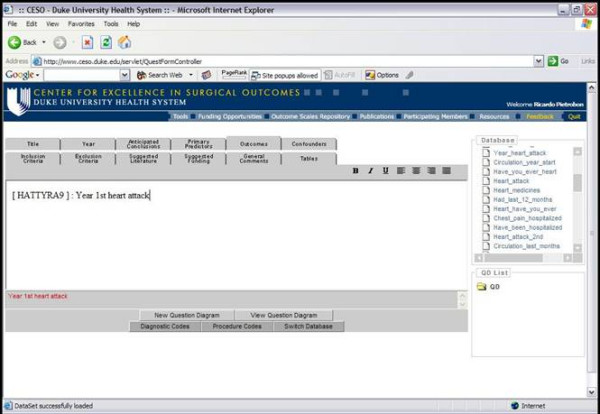
QuestForm application – Formulation of a Question Diagram

**Figure 5:**
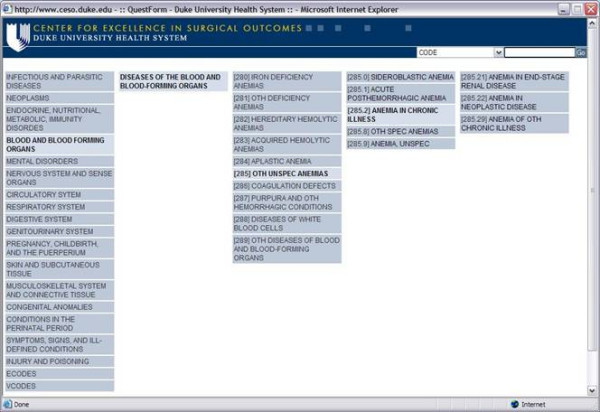
QuestForm application – Search engine for ICD9 codes

QuestForm starts by presenting researchers with three main strategies to find research databases: use of pre-determined key words that describe the database as a unit (Figure 2), user-defined key words to describe variables present in the data dictionary of each database, and the presentation of a complete list of all databases. Once databases are located, researchers can read an overall summary about the database including details about number of subjects, sampling strategy, data ownership, and overall characteristics of the study population and associated procedures (Figure 3). Researchers then determine that the database most appropriate to answer the research question at hand, a JAVA screen is displayed for research question formulation (Figure 4). This screen presents all variables displayed in hierarchical categories. Variables are presented with the corresponding question and alternative responses. All variables can be inserted into a research question (Question Diagram) divided into the classical categories for an epidemiological question: Outcomes, Predictors, Confounders, Inclusion and Exclusion Criteria. Search engines are provided for ICD9-CM diagnosis and procedure codes (Figure 5), which can also be inserted into the Question Diagram. Finally, previously formulated Question Diagrams can be shared among researchers. This latter functionality allows researchers to both share Question Diagrams among members of the ongoing project as well as share previously formulated Question Diagrams with researchers from other teams. Once the question is fully formulated, researchers can save the question as in a graphical format known as Question Diagram.

#### Outcomes research application

Dr. Guller initiated the project searching for an existing database that would allow him to compare surgical outcomes between laparoscopic and open appendectomy procedures in the treatment of acute appendicitis. The outcomes were pre-specified as mortality and infection, although other existing outcomes would also be of interest. Although there are multiple single-institution studies attempting to answer this question [6,7], few studies have taken a population approach to test whether one procedure is superior to the other.

As a first step, Dr. Guller searched across previously formulated Question Diagrams to evaluate whether other studies could have used a similar research design. Since none was found in QuestForm, Dr. Guller searched across more than forty different databases for an existing database that would have the variables to answer his research question. After navigating through multiple data dictionaries, Dr. Guller found that the Nationwide Inpatient Sample (NIS) Release 6, 1997 [8] presented the variables and an adequate number of patients to answer his question.

Once the database was located, Dr. Guller selected the outcomes of interest (length of hospital stay, in-hospital complications, in-hospital mortality, rate of routine discharge), main predictors (laparoscopic versus open procedures), and confounders (age, gender, race, household income, comorbidity, hospital volume, location of the hospital, teaching status of hospital, and appendix perforation), inserting each of them into the research question fields in QuestForm. Using built-in search engines for ICD9 codes, Dr. Guller created the definition for each of the above-mentioned variables and defined the inclusion and exclusion criteria. The final research question was then saved as a Question Diagram and immediately submitted to Dr. Pietrobon for feasibility evaluation. Dr. Pietrobon judged that the project was feasible and could be completed using the database indicated by Dr. Guller. At this point, the project was initiated and a detailed project management plan was established using Research Manager.

### Research manager

Research Manager is a Web application developed by our group designed to facilitate the project management of clinical research projects. Similar to QuestForm, Research Manager is licensed under the GNU Public License [10], which allows individuals to copy, modify, and freely distribute the software as long as the source code is provided.

**Figure 6:**
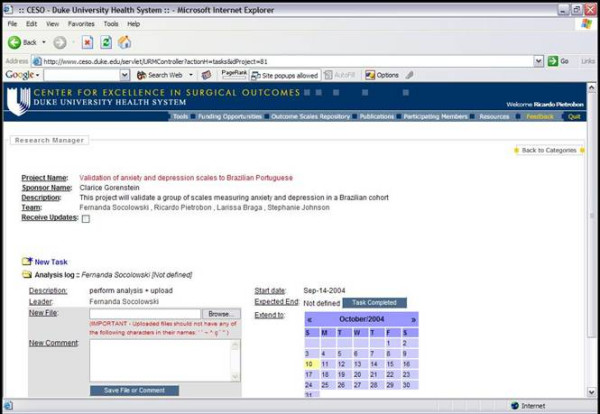
Research Manager

Research Manager provides multiple features to facilitate project management of clinical research projects. All projects are displayed by category (e.g., cardiology, general surgery, etc) with a brief description. The internal content of all projects is password protected. All internal tasks within a project are assigned to individual researchers. Project administrators initially assign deadlines that can be modified by task leaders within three days. All participating members of the project receive weekly reports containing details about the activity and the latest electronic file within each task (Figure 6). These files can include research questions, data analysis files, synthesis of a literature review, and manuscript drafts. Project members can also customize the application to receive updates for every single file uploaded to Research Manager in real time if they decide to closely track the project. Expired tasks are marked in the weekly report sent to the entire team, thus providing an incentive for investigators to keep tasks within planned deadlines.

Research Manager helps identifying such problems and enables their early elimination, thus avoiding delays in the project completion. Finally, since weekly reports are generated to all participating members, Research Manager also provides peer-incentive for project members to complete their tasks in a timely manner.

#### Outcomes research application

Once the Question Diagram was evaluated by the clinical epidemiologist, this project was transferred to Research Manager. Contact information for each of the researchers involved and deadlines for completion of the main phases of the research process were set, including data extraction and cleaning, data analysis, literature review, and manuscript writing. Weekly reports were generated to update investigators on all tasks of the project, including different versions of the statistical analysis, modifications in the research question, and manuscript sections.

With an established project plan, the statistician in charge selected the best methods for analysis. Since the database is a random sample of the United States and requires special survey analysis methods, it was necessary that all involved researchers understood the statistical approach by using Analysis Charts.

### Analysis charts

**Figure 7:**
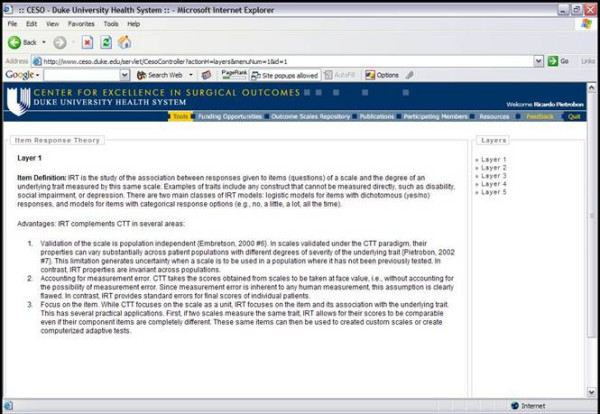
Analysis Chart

Analysis Chart is a tool designed to enhance the understanding of statistical methods to a format that is understandable by clinical researchers with different previous levels of statistical knowledge. As such, it is important in the design as well as the analysis phases of a project (Figure 7). The application was built using Extensible HyperText Markup Language (XHTML 1.0) [4] in combination with Cascading Style Sheets [11].

Analysis charts are composed by cascading links that display information about quantitative methods in progressive levels of complexity called "layers of information". Each layer explains the statistical method with an increasing level of complexity. In this manner, clinicians interested in simply understanding the method to evaluate whether it can be applied to a research project can simply read the first three layers. In contrast, researchers interested in a direct application of the method to available research data can follow all layers and their respective references. Most commonly, complex techniques are presented using five *layers of information*. Layer 1 summarizes the general goal of the method. Layer 2 presents previous clinical applications so that the researcher can visualize situations in which the method may be realistically applied. Layer 3 describes the data requirements for the application of the statistical technique. Layer 4 describes the basic statistical underpinnings of the method, initially breaking down equations and then reassembling them. Finally, layer 5 presents a list of available software packages for the implementation of the technique as well as cases studies where all previous layers are applied to real data sets. Each layer ends with a section containing selected references that explain the topic in more detail.

#### Outcomes research application

While deciding on the most appropriate analysis strategy for the Question Diagram, Drs. Pietrobon and Guller consulted the Analysis Chart searching for the most appropriate statistical methods of analysis. Given the nature of the research question and that the NIS database has a sample design, Drs. Guller and Pietrobon opted for an approach involving multiple and logistic regression models while adjusting for sampling weights, strata, and clusters. With a defined analysis protocol, the research question was then transferred to a statistical programmer trained in the translation of Question Diagrams into statistical code. This process was closely evaluated by Drs. Pietrobon and Guller, who scrutinized the statistical code and results from a clinical and statistical perspectives. Once the results were deemed to be accurate, Dr. Pietrobon started the literature review using a Literature Matrix.

### Literature matrices

**Figure 8:**
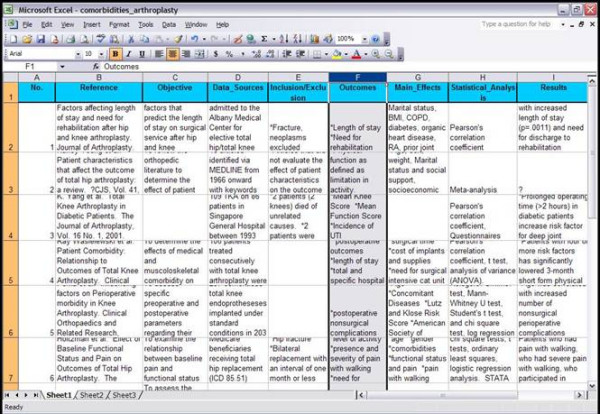
Literature Matrix

Literature matrices consist of a comprehensive but not necessarily exhaustive review of the literature focused on a narrow clinical topic (Figure 8). Each article is analyzed using the following criteria: study objectives, data sources, outcome variables, primary predictor variables, confounders, statistical analysis, results, established knowledge, and shortcomings. Each literature matrix is saved as an XHTML file that can be visualized in web browsers as well as imported in any commercial or open source spreadsheet applications such as Microsoft Excel^® ^or Open Office Calc [12].

The advantage of the Literature Matrix as implemented in the Web suite is its availability over the web to the whole outcomes research community. This allows Literature Matrices to be constantly updated, with authors receiving their due credit in a list of contributors. Literature Matrices also enable researchers to obtain a complete summary of the literature without going through the cumbersome process of copying and reading a manuscript for the first time. In contrast, researchers' time can be spent more efficiently in reviewing what has already been compiled and attempting to expand the Literature Matrix with other relevant bibliographic references.

#### Outcomes research application

In order to evaluate the literature, a graduate student performed a thorough literature search. All relevant articles were copied, read, and the data extracted according to the established categories. Once the Literature Matrix had been completed, Drs. Guller compared the current project results with results published in the literature. The structured information in Literature Matrices also allowed Dr. Guller to compare the strengths and weaknesses of the current project in relation to previous publications. Once this phase was completed, Dr. Guller proceeded to the final writing of the manuscript using Output Templates.

#### Outcomes research application

At the end of the project, Dr. Guller combined the Question Diagram, Analysis Chart, and Literature Matrices to write the final manuscript. While Analysis Charts provided the information concerning the statistical techniques used in this study, Literature Matrices provided the basis for comparison of the study results against previous publications. Although not included in the final manuscript, Dr. Guller also had access to multiple analysis files through Research Manager to orient him in each of the steps taken during the research question formulation, data analysis, and literature review.

### Use of web application by clinical researchers

**Figure 9:**
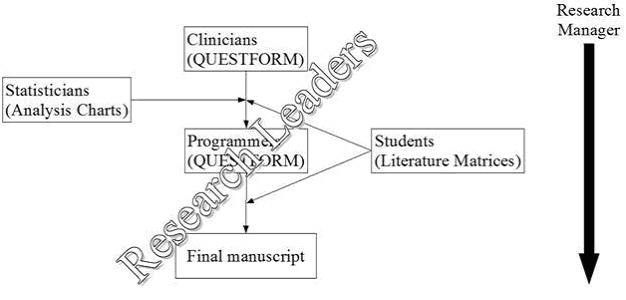
Integration between UR tools and clinical researchers

The use of web applications by individual clinical researchers can be summarized in Figure 9.

## Utility

Since the concept of streamlining the interdisciplinary collaboration preceded the existence of the current suite of web applications, different versions of the central idea have been gradually applied since the second half of 2002. Although our usability, qualitative, and economic studies to evaluate this application are still ongoing, we have noticed a significant improvement in the number and quality of our publications as evidenced by the increasing acceptance rates of our manuscripts for publication in peer-reviewed journals. The Web applications are currently used in research projects involving Duke University and other universities in the United States and abroad, where they have been shown to facilitate inter-institutional collaborations.

## Discussion

Improving the efficiency of an interdisciplinary approach to secondary data analyses has multiple potential benefits. These include an increase in the overall clinical significance of the final publication, decrease in the number of failed projects, decrease in the time for completion of individual projects, improvement in the education of future outcomes researchers, decrease in the cost-benefit ratio for individual outcomes research projects, and, perhaps most importantly, an automation of repetitive tasks. This last factor is crucial since eliminating repetitive tasks will allow researchers to concentrate on the design of innovative projects [2].

Surprisingly, few systems and applications have been described to solve the problem of complex interdisciplinary collaboration between clinicians and statisticians. Isolated approaches have usually focused on specific portions of the outcomes research process, without attempting to integrate them into a cohesive system. For example, Marshall [13] has proposed the use of a secure Internet web site for collaborative medical research and data collection. While this system seems to achieve its proposed objectives, it does not improve the process of guiding teams in the translation of data into useful clinical information. Other systems have approached the process in a more comprehensive manner. The Research Toolbox [14], for example, is a software application that combines databases for literature searches in addition to providing templates for the scientific output. The system is applicable to any type of research, but lacks the ability to connect researchers over the World Wide Web. It also does not address the formulation of research questions from existing databases, selection of statistical techniques, exchange of manuscripts, or project management. Finally, the Web-based Medical Information Retrieval System (WebMIRS) project, funded by the National Library of Medicine [15], allows researchers not only to evaluate the database content but also to perform the data extraction of specific subsets of the data set. In spite of its high performance as a research tool, WebMIRS is currently restricted to one single publicly available database (National Health and Examination Survey – NHANES), and does not contribute to other phases of the outcomes research process.

Although this newly developed system of applications provides a significant improvement in the way secondary data analyses are conducted, it still has limitations. First, because of the lack of a formal evaluation of the effectiveness of this system, we are unable to quantify its real time and cost saving benefits. Second, the system is currently restricted to secondary analyses and does not allow for the planning of prospective data collection. Although one of the main advantages of formulating a research question based on existing data sets is the bounded nature of the process, future applications should attempt to create rules and algorithms that may guide prospective data collection.

## Conclusion

In summary, we have experienced that this system has significant advantages over the traditional manner of conducting outcomes research based on secondary data analyses. This tool may have important applications, not only resulting in an improvement in the overall efficiency of the outcomes research process, but also affecting the way new outcomes researchers are trained and introduced to a research environment.

## Availability and requirements

The Web application is available at 

## Abbreviations

XHTML Extensible HyperText Markup Language 1.0

JAVA: by Sun Microsystems

ICD9-CM: International Classification of Diseases, Ninth Revision – Clinical Modification

NIS: Nationwide Inpatient Sample

GNU: GNU's Not linux General Public License

WebMIRS: Web-based Medical Information Retrieval System

NHANES: National Health and Examination Survey

UR: Uniform Resource

## Competing interests

The author(s) declare that they have no competing interests

## Authors' contributions

Ricardo Pietrobon – design, manuscript drafting

Ulrich Guller – design, manuscript revision for important intellectual content

Henrique Martins – design, manuscript revision for important intellectual content, software programming

Andreia P Menezes – design, manuscript revision for important intellectual content

Laurence D. Higgins – design, manuscript revision for important intellectual content

Danny O. Jacobs – design, manuscript revision for important intellectual content

## Pre-publication history

The pre-publication history for this paper can be accessed here:


